# Awareness, Willingness and Use of HIV Pre-Exposure Prophylaxis Among Female Sex Workers Living in Dar-es-Salaam, Tanzania

**DOI:** 10.1007/s10461-022-03769-4

**Published:** 2022-07-15

**Authors:** Diana Faini, Patricia Munseri, Eric Sandstrom, Claudia Hanson, Muhammad Bakari

**Affiliations:** 1 Department of Epidemiology and Biostatistics, Muhimbili University of Health and Allied Sciences (MUHAS), 9 United Nations Road, Dar es Salaam, Tanzania; 2 Department of Global Public Health—Health Systems and Policy, Karolinska Institutet, Stockholm, Sweden; 3 Department of Internal Medicine, Muhimbili University of Health and Allied Sciences (MUHAS), Dar es Salaam, Tanzania; 4 Department of Disease Control, London School of Hygiene and Tropical Medicine, London, UK

**Keywords:** Pre-exposure prophylaxis-PrEP, Female-sex workers, HIV prevention, Tanzania

## Abstract

Tanzania is in the final stages to roll out pre-exposure prophylaxis (PrEP) to Female Sex Workers (FSWs) so as to reduce new infections. PrEP demonstration projects support programming through gaining first experiences.

We analyzed data from a cohort of 700 HIV negative FSWs in Dar-es-Salaam to determine proportions of FSWs who were aware, willing and used PrEP. We compared proportions at cohort enrolment and after 12 months. Logistic regression was used to determine factors associated with PrEP use. PrEP awareness increased from 67% to 97% after 12 months. Willingness was high at both time points (98% versus 96%). Only 8% (57/700) had used PrEP. Being married/cohabiting or separated/divorced/widowed and having sex with a HIV infected partner were independently associated with PrEP use. The PrEP program should focus on scaling up access as willingness to use PrEP is high.

## Introduction

In the past decade, several clinical trials have demonstrated that the use of antiretroviral drugs as pre-exposure prophylaxis (PrEP), is effective and safe for preventing HIV infection (1–3). In response, in the year 2015 the World Health Organization recommended PrEP to be used among populations who have a HIV incidence greater than 3 per 100 person years of observation (4). Although PrEP is increasingly recognized as a critical component of comprehensive HIV programmes, the scale-up of PrEP delivery to high-risk populations has been slow in many countries in sub-Saharan Africa including Tanzania (5).

Female sex workers (FSWs) are recognized as a population at an increased risk of HIV infection in Tanzania (6–8). HIV incidence in this population has been reported to be above 3 per 100 person years in different regions within the country (9–12). The high HIV burden among FSWs has been attributed to several factors including, the inability to insist on consistent condom use and sexual violence because of gender power imbalances (13–17). PrEP offers FSWs a promising method of protection against the risk of HIV acquisition which they can initiate and control on their own (3, 18, 19).

Published literature from studies in Tanzania have reported high PrEP interest but also expressed concerns around cost, readiness of health care providers, adherence, side-effects, and stigma related to using PrEP (20–24). However, available literature has not reported on the extent of awareness, willingness, and use of PrEP among FSWs. PrEP is still a relatively new biomedical intervention and therefore understanding PrEP uptake and its determinants is necessary to ensure that the future PrEP program supports proper and consistent PrEP use.

In this paper, we report on awareness, willingness, and use of PrEP among FSWs recruited for participation in a vaccine preparedness cohort in Dar es Salaam. At the time of data collection for this study, PrEP was available through a PrEP demonstration project implemented by the ministry responsible for health as well as research studies conducted in the city (25). We report on the number of FSWs enrolled in our cohort reporting to have ever initiated PrEP at any point during their one-year follow-up. We also report on their awareness and willingness to use PrEP at the time of enrolment and changes after 12-months of follow-up. This paper considers PrEP as a new intervention within the context of impending PrEP rollout programme in Tanzania.

**Methods.**

### Study Design and Study Population

We performed a cross-sectional analysis of data from a cohort study—the PrEPVacc registration cohort. The PrEPVacc registration cohort has been described previously (12, 26, 27). In brief, FSWs living in Dar es Salaam were recruited through respondent driven sampling conducted from October 2018 to January 2019. Potential participants with valid recruitment coupons were provided with study information and procedures in *Kiswahili*. Those willing to participate signed an informed consent form before undergoing any study procedures. Thereafter, participants were, enrolled and followed-up to determine their HIV incidence in preparation for a phase IIb multi-centre HIV vaccine trial (PrEPVacc trial)^26^. FSWs were eligible for enrolment into the cohort if they were aged 18–45 years, had a negative HIV antibody test, considered to be at increased risk for HIV infection and resided in Dar es Salaam. After enrollment, they were followed every three months for assessment of HIV risk behaviours and collection of blood samples for HIV testing. The participants were assessed on their awareness, willingness, and use of PrEP at their enrollment, and at sixth- and 12-months visits. Follow up data included in this analysis were collected from January 2019 to January 2020.

### Access to PrEP and PrEP education provided in the study

PrEP in Tanzania was made available in early 2018 through the PrEP demonstration project funded by PEPFAR and USAID (25). Apart from the PrEP demonstration project, a number of research projects have been offering PrEP as early as 2011 to most at risk populations, including adolescent girls and young women (aged 16–24 years), sero-discordant couples, female bar workers, FSWs and partners of FSWs (20–23, 25, 28, 29). As of October 2020, there were cumulatively 18,000–19,000 estimated PrEP users in Tanzania (25).

PrEP was not offered at the PrEPVacc study site during the time of data collection. This is because PrEP was not widely available in Tanzania during the study period. Participants were encouraged to apply for PrEP through the available channels such as the PrEP demonstration project and other research projects offering PrEP within the FSWs community. Cohort participants were informed that PrEP (Descovy© and Truvada©) would be offered in the future at the study site through the PrEPVacc HIV vaccine trial.

Study nurses and doctors who had received training on PrEP, provided information to each participant on the benefits of PrEP when taken consistently. They also explained side effects of using PrEP such as tiredness and headaches. PrEP education was offered during the enrollment visit, as well as the sixth- and 12-months visits. Additionally, PrEP education was provided to groups of participants in seminars conducted weekly as part of cohort engagement activities. In these seminars, participants were encouraged to ask questions related to PrEP and myths or misinformations regarding on PrEP were addressed.

**Study Measures**.

A questionnaire administered by study nurses and doctors at enrollment, 6 months and 12 months visits were used to assess PrEP awareness, PrEP use, and willingness to use PrEP. First, participants were assessed on PrEP awareness with a Yes/No response to the question “Have you heard about pre-exposure prophylaxis (PrEP) i.e the use of anti-HIV drugs by HIV-negative persons to protect themselves from catching HIV?”. We categorized those responding “Yes” for each of the three visits (enrollment, 6 months or 12 months) as “Aware of PrEP”. In the analysis we compared proportions of FSWs who were aware of PrEP at the enrolment visit to the proportions at the 12-months visit. Second, participants who were aware of PrEP were assessed on PrEP use with a Yes/No response to the question “Are you currently using PrEP”. In the analysis, we generated a variable “Ever used PrEP” which included all FSWs responding “Yes” in any of the three visits (enrollment, 6 months or 12 months), and the rest categorized as “Never used PrEP. Lastly, willingness to use PrEP was assessed using the question “Will you be willing to use PrEP if it were offered to you?” among participants who were not aware of PrEP and not using PrEP. Responses were recorded as “No”, “Yes” or “Not sure”. In the analysis, responses of “No” and “Not sure” to PrEP willingness were combined to “Not willing to use PrEP” and those remaining were categorized as “Willing to use PrEP”. We then compared proportions of PrEP willingness at the enrolment visit to that at the 12-months visit.

**Covariates.** We used demographic and sexual risk behaviours reported at the enrolment visit to examine factors associated with the main outcome— PrEP use. Variables were categorized based on their distribution so as to minimize data sparsity. Age was categorized into three groups i.e. “18–24 years”, “25–34 years” and “35–45 years”. Education level was grouped into three categories, (i) no formal education or incomplete primary education, (ii) completed seven years of primary education or some secondary education and (iii) completed secondary education together with college or any other post-secondary school training. Marital status was also categorized into three groups i.e. “Never married”, “Married or co-habiting with a male partner” and “Separated, divorced or widowed”. The number of reported sexual partners in the past three months was dichotomized at median value to “120 partners or less” or “more than 120 partners” whereas the number of new sexual partners during the past three months was dichotomized to “48 partners or less” or “49 partners and more”. All participants were screened for Syphilis, Hepatitis B and C virus infections prior to enrolment. A composite variable for sexually transmitted infections (STI) was generated by grouping those testing positive to at least one of the infections as “Positive” and those testing negative to all three STIs (Syphilis, Hepatitis B or C infection) as “Negative”. Other sexual risk behaviour variables (“condom use during transactional sex”, “condom use with a new sexual partner”, “condom use with a HIV infected partner”, “history of STI treatment” and “rape” in the past three months) were reported as binary or categorical variables as per responses recorded at the enrolment interview.

### Statistical analysis

The distribution of socio-demographic and behavioural variables was summarized as frequencies and proportions. This was done for the overall study population and separately for those reporting to have ever used PrEP and those who have never used PrEP. We compared proportions across the two groups using a Chi squared test for differences in proportions. Variables with differences in PrEP use at p < 0.05 were included in a multivariable logistic regression model to characterize associations. In building the multivariable logistic regression model, the “rule of ten” was used so as to ensure that covariate in the model did not exceed 10% of the primary outcome i.e. number of PrEP users.

We excluded colinear variables i.e. factors having the same meaning from the model. Thus, the covariate “Total number of partners” and “number of new sexual partners” were thought to be colinear and hence only one covariate was included in the model. The final multivariable model had the following covariates: marital status, number of partners in the last three months, sex with a HIV infected partner, condom usage during transactional sex and rape. Adjusted odds ratio estimates from the final model, 95% CI and the likelihood ratio test for statistical significance were reported. All analyses were conducted using STATA version 15.

Proportions of PrEP awareness and willingness at the enrollment visit and at 12 months were summarized. McNemar’s Chi-square test for paired proportion was used to compare the proportions at enrolment and at the 12-months visit. The paired analysis was restricted to participants who attended both visits.

### Ethical considerations

Ethical clearance for this analysis was obtained from the MUHAS Institutional Review Board (MUHAS IRB) with Ref. MUHAS-RE-4-2020-200. The PrEPVacc registration cohort study received ethical approval from MUHAS IRB with Ref. No. 2018-04-04/AEC/Vol. XII/335 and the National Health Research Ethics Committee (NatHREC) Ref. No. NIMR/HQ/R.8a/Vol.IX/2809.

## Results

### Socio demographic and risky sexual behavioural characteristics at enrolment

A total of 700 HIV negative female sex workers were enrolled in the PrEPVacc cohort. The median age (IQR) at enrollment was 25 years (IQR 21–32 years). More than half of the sex workers in the cohort had completed seven years of primary education (69%, 480/700) and the majority had never been married (85%, 594/700).

At cohort enrolment, more than half of the FSWs reported to have had more than 120 sexual partners (61%, 426/700) and had more than 48 new partners (52%, 361/700) within the three months preceding the enrollment interview. Only a few (6%, 42/700), reported to consistently use condoms during transactional sex, or when having sex with a new sexual partner (19%, 133/700). There were 120 FSWs (17%, 120/700) who reported to have been raped at least once within the three months preceding the interview. At enrolment, (3%, 22/700) were diagnosed with at least one sexually transmitted infection (Syphilis, Hepatitis B or Hepatitis C virus infection) whereas, (6%, 45/700) reported to have been diagnosed or received treatment for a sexually transmitted infection within three months preceding cohort enrollment.

### Awareness and willingness to use PrEP

At enrolment, (67%, 469/700) of FSWs reported to have ever heard of PrEP. This awareness of PrEP among the study participants increased over time, whereby at 12 months (97%, 545/562) reported to be aware of PrEP (McNemar Chi-squared p < 0.001, **Table **1).

Overall, there was high willingness to use PrEP among FSWs in the cohort. At enrolment, (98%, 684/700) of the participants said they were willing to use PrEP if it was offered. Of the remaining 16 participants, four reported not to be willing to use PrEP and 12 FSWs were not sure if they would use it. There was no statistical significant change in PrEP willingness after 12 months follow-up (98% vs. 96% McNemar test p = 0.84 **Table 1).** A sub-analysis of the data showed that, at enrolment, only 67% (460/684) of those willing to use PrEP, had previously heard of it compared to the 99% (534/541) at 12 months.

**Table 1:** PrEP awareness, willingness and use at enrolment and at 12-months

	At cohort enrolment n/N (%)	At 12 months follow up [1] n/N (%)	P-value [2]
Aware of PrEP	469/700(67%)	545/562(97%)	< 0.001
Willing to use PrEP	684/700(98%)	541/562(96%)	0.840
Willingness among those aware of PrEP	460/469(98%)	534/545(98%)	0.910

[1] Estimated among FSWs with follow-up data. [2] Mc-Nemar Chi-squared test for paired proportions

### PrEP use and determinants of PrEP use

A total of 57 HIV negative FSWs (8%, 57/700), reported to have ever initiated the use of PrEP at any point during the 12 months follow up period. Nearly all of the FSWs who had ever used PrEP (86%, 49/57), reported to have received PrEP from a Non-governmental organization (NGO). Other FSWs (11%, 6/57) reported to have had received PrEP from a friend – possibly a peer educator within the FSWs community, and the rest (4%, 2/57) received from a private or government clinic.

There were no age or education differences between FSWs who had ever used PrEP as compared to those who had never used it. A higher proportion of PrEP users were married/cohabiting or separated/divorced/widowed as compared to PrEP nonusers (9% vs. 3% and 21% vs. 11%, p = 0.002). PrEP users reported a higher number of sexual partners compared to non-PrEP users (44% vs. 38%, p = 0.002, **Table **2). PrEP users also reported more consistent condom use during transactional sex compared to PrEP non-users (11% vs. 6%, p < 0.001, **Table **2). On the other hand, PrEP users reported relatively more instances of having sex with a HIV infected partner (7% vs. 2%, p = 0.001, **Table **2) as compared to participants who had never used PrEP.

In the multivariable logistic regression model (**Table **3), PrEP use was adjusted for marital status, number of sexual partners, having sex with HIV infected partner, condom use and rape. We found that marital status was strongly associated with PrEP use whereby; FSWs who were married/co-habiting were four times more likely to use PrEP (aOR 4.19, 95% CI 1.44–12.18), while those separated /divorced /widowed were twice as likely to use PrEP (aOR 2.38, 95% CI 1.17–4.83) compared to FSWs who had never been married. Also, we found that FSWs who reported to have had sex with a HIV infected partner were associated with four times the odds of using PrEP (aOR 3.98, 95% CI 1.20- 13.15, p = 0.04). However, we did not find any statistical association between PrEP use with number of partners, consistent condom use during transactional sex or with experiences of rape.

**Table 2:** Demographic and behavioural characteristics

Characteristic	ALL (n, col %)	PrEP users n(col)	PrEP non-users n(col)	X^2^ p-value	
**Overall**	700(100%)	57(100)	643(100)		
**Age (years)**					
18–24	327(47)	26(46)	301(47)	0.303	
25–34	272(39)	19(33)	253 (39)		
35–45	101(14)	12(21)	89(14)		
**Education**					
None/Incomplete Primary education	83(12)	9(16)	74(12)	0.564	
Complete Primary/Incomplete secondary education	480(69)	36(63)	444(69)		
Complete secondary or higher	137(20)	12(21)	125(19)		
**Relationship status**					
Never Married	594(85)	40(70)	554(86)	0.002	
Married /Cohabiting	22(3)	5(9)	17(3)		
Separated/Divorced/Widowed	84(12)	12(21)	72(11)		
**Total number of partners in the past 3 months** [1]					
≤ 120	426(61)	31(54)	395(61)	0.002	
121+	273(39)	25(44)	248(38)		
**Number of new partners in the past 3 months** [1]					
≤ 48	361(52)	30(53)	104(16)	0.003	
49+	338(48)	26(46)	539(84)		
**Condom use during transactional sex** [2]					
Always	42(6)	6(11)	36(6)	< 0.001	
Sometimes	622(89)	47(83)	575(89)		
Never	34(5)	2(4)	32(5)		
**Sex without a condom with a NEW sex partner in past 3 months**					
No	133(19)	16(28)	117(18)	0.069	
Yes	567(81)	41(72)	526(82)		
**Sex with HIV infected partner in the past 3 months** [1]					
No	10(1)	1(2)	9(1)	0.001	
Yes	16(2)	4(7)	12(2)		
Don’t know	673(96)	51(90)	622(97)		
**History of STI treatment in the past 3 months**					
No	655(94)	53(93)	602(94)	0.850	
Yes	45(6)	4(7)	41(6)		
**STI diagnosis at enrolment**					
Negative	678(97)	55(96)	623(97)	0.869	
Positive	22(3)	2(4)	20(3)		
**Experience of rape in the past 3 months** [1]					
No	579(83)	42(74)	537(84)	0.001	
Yes	120(17)	14(25)	106(16)		
Totals may not add up due to missing values [1] 1 missing data.[2] Two missing values.					

**Table 3:** Multivariable analysis

Characteristic	FSWs who have EVER used PrEP/Subtotal (row %)	Adjusted OR	P-value
**Relationship status**			
Never Married	40/594(7)	1	0.01
Married /Cohabiting	5/22(23)	4.19(1.44–12.18)	
Separated/Divorced/Widowed	12/84(14)	2.38(1.17–4.83)	
**Number of partners in the past 3 months**			
≤ 120	31/426(7)	1	0.52
121+	25/273(9)	1.20(0.68–2.13)	
**Sex with HIV infected partner in the past 3 months**			
No/Don’t Know	52/683(8)	1	0.04
Yes	4/16(25)	3.98(1.20-13.15)	
**Condom use during transactional sex**			
Always	6/42(14)	1	0.34
Sometimes	47/622(8)	2.49(0.45–13.66)	
Never	2/34(6)	1.19(0.27–5.26)	
**Rape in the past 3 months**			
No	42/579(7)	1	0.15
Yes	14/120(12)	1.65(0.85–3.21)	

## Discussion

This analysis examined PrEP awareness, willingness and use among a cohort of HIV negative FSWs in Dar es Salaam, Tanzania. PrEP awareness and willingness among FSWs in the cohort was high at enrollment and over the 12 months follow-up period. However, we found that only 8% of the FSWs in the cohort had ever used PrEP, and among them the use was significantly higher among FSWs who were married/cohabiting, and those engaging in sex with a HIV infected partner.

We found that, at cohort enrolment more than half of the sex workers had ever heard of PrEP. This was higher than the 5% reported among female bar workers in Dar es Salaam who are known to sell sex to supplement their income, serving as “indirect” sex workers (22). This difference could be explained by the fact that, the PrEP demonstration project that was conducted by the ministry responsible for health ahead of our data collection period, had targeted “direct” sex workers whose primary mode of income is sex work.

The increase in PrEP awareness observed over the follow-up period was encouraging. This increase was likely attributed to the repeated PrEP education seminars which were offered in the cohort so as to increase PrEP awareness ahead of the PrEPVacc trial. Raising PrEP awareness is vital to PrEP initiation and is one of the first steps in engaging sex workers along the PrEP care continuum (30). Community engagement and public health campaigns on PrEP would also help raise PrEP awareness within communities and thus potentially increase uptake. We therefore believe that during implementation of the PrEP roll-out in the country, FSWs in our cohort may be resourceful and can serve as peer educators to promote PrEP uptake within their networks.

PrEP willingness in our study was encouragingly high. It was higher than the 60% reported among female bar workers (who also sold sex for money) in a study conducted in 2019 in Dar es Salaam (22). The PrEP willingness was also higher than the 61% reported among FSWs in Kenya and Uganda (31). The higher PrEP willingness observed in our study could be explained by the fact that, during recruitment, participants were informed that PrEP would be offered as part of the envisaged PrEPVacc trial. It is likely that those consenting to enroll into the PrEPVacc cohort already had interest in using PrEP. This is supported by the higher proportion of FSWs in our study who were aware of PrEP and a higher proportion of FSWs who had ever used PrEP compared to FSWs in the other studies. We also noted that, even though many FSWs quickly indicated their willingness to use PrEP as soon as they heard of it, willingness was only sustained after repeated educational sessions on PrEP. We believe that the PrEP education provided repeatedly to FSWs during their follow up visits increased their confidence and sustained their willingness to use PrEP. Therefore, repeated PrEP education will also be vital so as sustain PrEP adherence and retention in the Tanzanian PrEP program.

Very few FSWs reported to have initiated PrEP despite the high PrEP awareness and willingness. The low PrEP use could be explained by the fact that PrEP is fairly new in Tanzania, not widely available and the fact that even health care providers serving populations at high HIV risk have not been fully trained on PrEP (21). Additionally, most of the research projects that were offering PrEP during the time of our data collection mainly targeted sero-discordant couples, adolescent girls, and young adults (21, 23–25). Success of the PrEP program among FSWs will largely depend on PrEP accessibility. One possibility would be to provide PrEP in sex-worker friendly clinics, preferably integrated with contraceptive and/or HIV testing services. The integration of PrEP, contraceptive use and HIV services is widely recommended and has been shown to improve demand and uptake of PrEP (32–35). This is because the use of PrEP is associated with the stigma of being labelled HIV infected or promiscuous (21, 36, 37). Hence integrated services provided in a sex-worker friendly environment will help PrEP uptake.

PrEP use was higher among FSWs engaging in sex with a HIV infected partner. PrEP use among FSWs with more risky sexual behaviour practices may reflect their higher perception for HIV acquisition risk and the need to protect themselves. This finding was similar to that observed in PrEP studies among FSWs in other settings (31, 37, 38) and also support studies that have reported higher PrEP use and adherence among women in sero-discordant relationships (39–41). The finding further shows the helpfulness of PrEP as a woman-controlled HIV prevention intervention in instances where negotiating condom use with a partner (especially a regular or intimate partner) is not possible or fails (42–45). Another plausible explanation for this observation would be behaviour disinhibition as a FSWs may knowingly engage in sex with a HIV positive partner because of the assured protective effectiveness of PrEP. Of note, this analysis cannot establish a temporal association to indicate whether FSWs engaged in higher risk behaviour after initiating the use of PrEP. However, studies have reported that risk compensation is of limited concern among PrEP users and that the use of PrEP may increase risk perception and encourage safer sexual behaviour practices (2, 3, 18, 19, 29, 46). Long-acting PrEP tools such as Dapivirine vaginal rings, implants and injectable PrEP may be ideal for FSWs and could probably more easily be integrated into their lifestyle as they offer a self-controlled HIV prevention without the problems of daily pill taking such as incorrect use, skipping pills, or taking pills when intoxicated with alcohol.

We also noted that PrEP use was higher among married /cohabiting FSWs as well as those who reported to be separated/divorced/widowed. Findings from other studies have reported that primary HIV risk for FSWs is likely to be from their main/regular partners rather than their clients because of the low condom use with the former (38, 47–50). Therefore, findings from our study could possibly reflect the utility of PrEP in relationships where females are unable to negotiate condom use and in the likely event that their spouse/main partner also has other sexual partners. Conversely, it may indicate that married/cohabiting FSWs were afraid of seroconverting and infecting the male partner in her stable relationship. It is also possible that the high PrEP use among married/cohabiting FSWs in our cohort simply reflects that these FSWs were participating in research projects targeting and offering PrEP to sero-discordant couples (25).

This study has limitations. First, PrEP was not widely available in Tanzania at the time of data collection. Therefore, our estimate on PrEP use does not reflect an uptake of PrEP if it were to be widely available. Nevertheless, a systematic review has shown positive responses in studies evaluating hypothetical PrEP use were supported by studies examining actual use and uptake (18). Second, data were drawn from an observation cohort which had specific inclusion criteria and therefore, our findings cannot be generalized to FSWs in the entire country. Third, a peer chain referral sampling method respondent driven sampling was used to recruit participants. This sampling method may have resulted in enrolment of more well-informed FSWs and those have larger social networks. This particular group is likely to be of FSWs who have been continuously involved in sex worker intervention programs and those who are also likely to be “early adopters” of PrEP. Fourth, social desirability bias is of concern in this study as participants may have overreported on sexual risk behaviour for the purpose of gaining study eligibility and may have also overreported willingness to use PrEP so as to please the research staff.

Despite these limitations, we provide a first insight to the level of PrEP awareness, willingness and use among FSWs in Dar es Salaam who have a high HIV incidence. Our study complements previous work on PrEP by examining potential user’s awareness, willingness to use PrEP and provide insights to potential determinants of PrEP use among FSWs.

## Conclusions

Our study indicated that FSWs in Dar es Salaam are largely aware of PrEP and were willing to initiate PrEP, still use was low. This finding makes it paramount to intensify programming and make PrEP easily available to FSW.

## Data Availability

Data cannot be shared publicly due to ethical restrictions as it contains identifying and sensitive information. These restrictions have been imposed by the National Health Research Ethics Committee (NatHREC) hosted by the National Institute for Medical Research (NIMR) of Tanzania.
